# Evidence of a genetically driven metabolomic signature in actively inflamed Crohn’s disease

**DOI:** 10.1038/s41598-022-18178-9

**Published:** 2022-08-18

**Authors:** Enrico Mossotto, Joanna Boberska, James J. Ashton, Imogen S. Stafford, Guo Cheng, Jonathan Baker, Florina Borca, Hang T. T. Phan, Tracy F. Coelho, R. Mark Beattie, Sandrine P. Claus, Sarah Ennis

**Affiliations:** 1 Human Genetics and Genomic Medicine, Southampton General Hospital, University of Southampton, Duthie Building (Mailpoint 808), Southampton, SO16 6YD UK; 2 Institute for Life Sciences, University of Southampton, Southampton, UK; 3 NIHR Southampton Biomedical Research Centre, University Hospital Southampton, Southampton, UK; 4 Department of Food and Nutritional Sciences, The University of Reading, Whiteknights campus, Reading, UK; 5 Department of Paediatric Gastroenterology, Southampton Children’s Hospital, Southampton, UK

**Keywords:** Genomics, Molecular medicine, Inflammatory bowel disease

## Abstract

Crohn’s disease (CD) is characterised by chronic inflammation. We aimed to identify a relationship between plasma inflammatory metabolomic signature and genomic data in CD using blood plasma metabolic profiles. Proton NMR spectroscopy were achieved for 228 paediatric CD patients. Regression (OPLS) modelling and machine learning (ML) approaches were independently applied to establish the metabolic inflammatory signature, which was correlated against gene-level pathogenicity scores generated for all patients and functional enrichment was analysed. OPLS modelling of metabolomic spectra from unfasted patients revealed distinctive shifts in plasma metabolites corresponding to regions of the spectrum assigned to *N*-acetyl glycoprotein, glycerol and phenylalanine that were highly correlated (R^2^ = 0.62) with C-reactive protein levels. The same metabolomic signature was independently identified using ML to predict patient inflammation status. Correlation of the individual peaks comprising this metabolomic signature of inflammation with pathogenic burden across 15,854 unselected genes identified significant enrichment for genes functioning within ‘intrinsic component of membrane’ (*p* = 0.003) and ‘inflammatory bowel disease (IBD)’ (*p* = 0.003). The seven genes contributing IBD enrichment are critical regulators of pro-inflammatory signaling. Overall, a metabolomic signature of inflammation can be detected from blood plasma in CD. This signal is correlated with pathogenic mutation in pro-inflammatory immune response genes.

## Introduction

Crohn’s disease (CD), one of the major subtypes of inflammatory bowel disease (IBD), is a heterogenous, relapsing, remitting condition characterised by transmural inflammation across the gastrointestinal tract. Disease aetiology centres on complex interaction between genetic predispostion and intestinal microbial exposure. Over 240 genes associated with IBD are enriched for proteins linked with bacterial recognition and response pathways, epithelial barrier integrity and downstream inflammatory signalling^1,2^. Whilst effective therapies exist, there is a clear need to stratify patients into risk groups for disease severity, complications and medication response. Reliable genetic and plasma biomarkers provide an attractive mechanism to stratify patients at diagnosis and during follow-up, whilst promoting novel drug discovery^3^.

Nuclear magnetic resonance (NMR) spectroscopy identifies precise constituents of biological samples, whereby molecules present distinct characteristic spectra. NMR has demonstrated the ability to discriminate IBD patients from controls through identification of dysregulated urine and plasma metabolites^4^; and distinguished IBD patients with active disease from those in remission^5^. Identification of genomic variation associated with disease severity markers, or biomarker profiles, can lead to targeted therapeutics and repurposing of known medications for new conditions^6^. Combining urine NMR spectra analysis with common variants identified through genome-wide association studies has previously been used to discover genetically determined metabolites in unselected samples^7^.

This study aimed to establish the discrete regions of the plasma metabolomic spectrum that specifically associate with inflammation measured using C-reactive protein (CRP) in paediatric CD patients. Following identification of a robust metabolomic signature of inflammation, we further aimed to compress the data underlying these discrete metabolomic peaks for correlation against exome sequencing data in order to identify genes and molecular pathways harbouring genetic variation that may explain altered the plasma metabolites. Ultimately, we wished to see how integrating metabolomic and genomic data could be used to stratify patients and inform therapeutic targeting.

## Materials and methods

### Patient samples

Patients aged < 18 years diagnosed with Crohn’s disease using the modified Porto criteria were recruited as part of the Genetics of IBD research study. Research blood samples were acquired during routine clinics.

Metabonomic and whole exome sequencing data were generated for a total of 228 patients diagnosed with CD. Where routine blood tests were clinically-requested on the same day as the plasma sample used for metabolomic analysis was acquired, these data were digitally retrieved from hospital records as previously described^8^. C-reactive protein (CRP) level was applied as our outcome measure to identify patients with actively inflamed disease. Patient medications at the time of plasma sampling were retrieved from electronic health records.

The study has ethics approval from Southampton & South West Hampshire Research Ethics Committee (09/H0504/125) and the study was conducted in accordance with relevant guidelines and regulations. All patients, or their parents/guardians, gave informed consent for participation in this study.

### DNA and plasma extraction

Genomic DNA was extracted from peripheral venous blood samples collected in Ethylenediaminetetraacetic acid (EDTA) using the salting out method. Deoxyribonucleic acid (DNA) concentration was estimated using the Qubit^®^ 2.0 Fluorometer and 260:280 ratio calculated using a nanodrop spectrophotometer. The average DNA yield obtained was 150 µg/ml and approximately 20ug of each patient DNA was extracted for next generation sequencing.

Plasma was isolated from peripheral venous blood by centrifuging samples for 10 min at 2000 RPM and 4 °C. After centrifugation, the plasma laying above the buffy coat was extracted and immediately frozen and stored at − 80 °C.

### Genomic data processing

Whole exome sequencing data were generated using Agilent SureSelect exon capture kits and Illumina HiSeq sequencing platforms. Processing and targeted analyses of the whole exome sequencing data applied herein have been presented elsewhere^9,10^. Genomic data were transformed into per-patient gene pathogenicity scores using the GenePy algorithm^11^. GenePy integrates the effect of multiple variants in each gene incorporating information on variant zygosity, frequency and deleteriousness (inferred using CADD v1.5 scores^12^). GenePy scores were initially generated for all patients for all 19,229 RefSeq genes. Genes with a Gene Damage Index (GDI) above the recommended threshold (GDI_Phred > 13.84) were excluded as genes with values above this level are considered highly mutable but unlikely to be disease causing^13^. This resulted in a final matrix of 15,854 GenePy scores for all patients.

### Metabolomics analysis of plasma

Plasma samples (200 µL) were mixed with deuterium water (D_2_O) (400 µL). The homogenized samples were centrifuged (10 min; 4 °C; 12,000 × *g*) and transferred to 5 mm NMR tubes for analysis by NMR spectroscopy. Plasma samples were processed into a single batch of 228 CD samples. NMR experiments applied a Bruker AV700 NMR instrument equipped with a 5 mm inverse CryoProbe^™^. A standard 1-dimensional NOESY-PR-1D experiment was performed on each sample, using a standard preset pulse sequence (noesypr1d90°). A Carr-Purcell-Meiboom-Gill (CPMG) experiment was applied (preset pulse sequence cpmgpr1d90°), where simple presaturation of the water signal was used. This experiment reduces the signal contribution from albumin and lipoproteins present in plasma and highlight signals from otherwise overshadowed smaller molecules. All samples were analysed at 297° K, 65 k data point spectrum (spectral width 19,607 Hz) was obtained by recording 256 scans (8 dummy scans). Phase and baseline of the spectra were corrected using MestreNova software v10.0m. NMR spectra were referenced to the glucose peak at δ 5.223 ppm.

### Metabolomics statistical analysis

Full resolution spectra were processed using Matlab vR2017a. The residual water signal was removed. Relative spectra were mean-centred and scaled to unit variance. Principal component analysis (PCA) was used to compare samples and identify outliers. Orthogonal Projection to Latent Structure (OPLS) analysis was performed for the supervised stage of the analysis, where NMR spectra were used as a matrix of variables. Regression of continuous patient CRP measurements against their metabolome data matrix, assessed plasma metabolic profile alteration with active inflammation. Model prediction was evaluated using goodness-of-fit correlation coefficient R^2^, showing what percentage of variation is explained by the model, and goodness-of-prediction (Q^2^), constituting the percentage of that variance which can be predicted by sevenfold cross-validation (hence splitting the input data in 7 subsets and recursively fit the model on 6 subsets and test its performance on 1 the left-out subset until all subset are used as test-set). Loadings were presented as a pseudo-NMR spectrum, plotting the model back-scaled coefficients and the weight of the variables. Metabolites with an R^2^ weight > 0.4 were considered highly discriminatory^14^.

### Machine learning classification

A random forest classifier (RF) of metabolic profiles was employed to predict patients with active inflammation as measured by CRP levels. While the metabolomic analysis utilised continuous CRP values to identify highly correlating peaks, the objective of the RF was to discriminate patients with negligible active inflammation from those with moderate/severe inflammation. Therefore, continuous CRP levels were binarised following the current WHO and FDA guidelines to classify patient bloods as either inflamed (CRP ≥ 5 mg/L) or non-inflamed (CRP < 5 mg/L)^15,16^.

**Figure 1:**
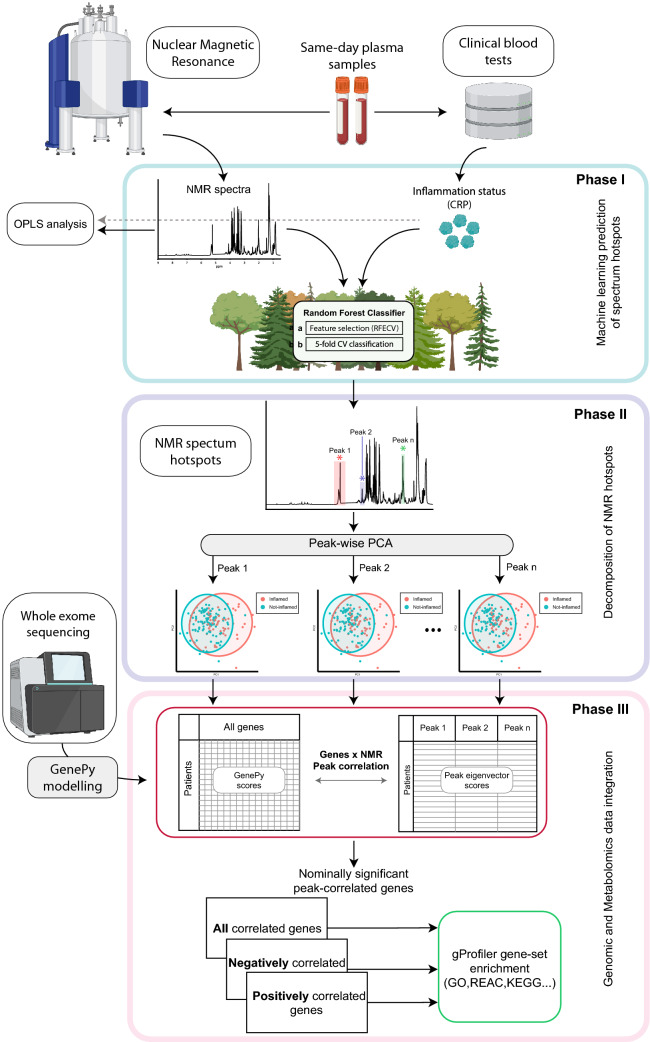
NMR and genomic data integration. Phase (I) NMR spectra and patient CRP data were input to the RF model using (a) RFECV and (b) cross-validated methods to select spectral regions discriminating non/inflamed patients. Phase (II) Informative data-points were clustered and peaks reduced to a single eigenvector. Phase (III) Eigenvectors for each peak were individually correlated against all genes and tested for enrichment. Created with BioRender.com.

The machine learning (ML) approach consisted of three phases (Fig. 1). The first phase involved the use of an RF classifier and a fivefold cross-validated recursive feature elimination approach (RFE-CV) to identify the regions of the NMR spectrum contributing to the non/inflamed patient classification (feature selection). This step recursively excludes 1% of the 38,470 datapoints comprising the metabolic profiles, until all the features are removed, and identifies datapoints consistently important for classification. The resultant selected regions were then employed to generate the final fivefold cross-validated RF model. Averaged metrics collected to assess performance include the F-1 statistic, precision, recall and balanced accuracy. From this final cross-validated model, features ranked within the top 5th percentile of importance were retained for further analysis.

The selected points of the spectra were subsequently binned by their location on the NMR spectrum. Groups of ≥ 10 points observed in close proximity were defined as ‘peaks’ and their constituent variance summarised using PCA. The discriminatory power of each component in separating inflamed/non-inflamed patients was assessed using Wilcoxon rank sum test and *p* values adjusted using false discovery rate (FDR). Components with a corrected *p* value < 0.05 were combined by their sum, generating a single eigenvector for each peak. This process transformed the multiple points within each of the *n* peaks into a matrix of eigenvector scores for each patient (Fig. 1, phase II). Summing those components significantly discriminating the inflamed and non-inflamed classes after FDR correction allowed for integration into downstream analyses.

In phase III, the resulting eigenvector matrix was integrated with the GenePy-transformed genomic data. These steps resulted in two matrices for all patients summarising: (1) eigenvector scores representing the metabolomic data most discriminatory of inflammation status and; (2) genetic data summarising the pathogenic burden of mutation for each gene. Spearman’s rank was used to correlate each of the metabolomic eigenvectors against GenePy scores for 15,854 genes. Genes with a nominally significant correlation were tested for enrichment in human databases (Gene Ontology, KEGG pathways, REACTOME, Complexes (CORUM), Human Phenotypes (HPA), WikiPathway (WP) using gProfiler2^17^. Enrichment scores (*p* values) were corrected using the SCS method embedded in the gProfiler2 model.

ML methods were applied using the Scikit-learn Python v3.7 library and R v4.0.3 packages.

## Results

Patients were recruited during routine clinics and untargeted with respect to diseases state, duration or treatment. As expected for paediatric Crohn’s disease, our cohort is characterised by an excess of male patients. The cohort reflected heterogeneity expected within clinical service with respect to time since diagnosis, disease state and treatment. Retrospective interrogation of clinical records identified 30 children who had undergone (24-h liquid diet and 4-h) fasting in preparation for colonoscopy and 154 patients for whom same-day blood tests had been clinically requested (Table 1). Medication data were available for all patients, supplementary data 1. Twenty-seven of the 154 patients were on no medications, or only nutritional therapy, at the time of plasma sampling, Table 2.

**Table 1:** Demographic and blood result data.

Clinical data		Inflamed	Non-inflamed
Number of samples	228	58	96
% Caucasian	93.0	97.6%	91.2%
% Male	71.2	75.9%	66.7%
Age in years at plasma extraction	14.0 (2.6–17.9)	14.0 (5.4–17.2)	14.1 (10.3–16.1)
Age in years at diagnosis	12.2 (1.3–16.9)	12.6 (4.1–16.1)	11.9 (2.4–16.6)
Time in years since diagnosis to point of sampling	1.8 (0.0–16.1)	1.4 (0.0–6.5)	2.2 (0.0–9.4)
Fasted (% of samples)	30 (13%)	0	0
CRP (mg/L)	8.75 (0–155)	20.1 (5–155)	1.1 (0–4)
ALB (g/L)	38.4 (23–51)	35.4 (25–45)	40.5 (26–51)
ESR (mm/h)	14 (1–68)	21.6 (5–68)	9 (1–41)
HB (g/L)	123.3 (73–166)	118.6 (89–144)	126.5 (80–166)
PCV (%/L)	0.4 (0.2–0.5)	0.35 (0.3–0.4)	0.4 (0.3–0.5)
PLT (10^9^/L)	343.5 (138–1018)	382 (148–1018)	316.3 (138–568)
WBC (10^9^/L)	7.6 (3.1–20.3)	8.4 (3.5–16.7)	7.1 (3.1–17.1)

Mean value is shown with (minimum–maximum) Ancestry was inferred from genomic data.

**Table 2:** Medication usage between inflamed and uninflamed patient groups.

	Thiopurine	Anti-TNF (infliximab or adalimumab)	Steroids	Exclusive enteral nutrition	Ustekinumab	Vedolizumab
CRP ≥ 5 (n = 58)	27 patients	6 patients	5 patients	7 patients	0 patients	0 patients
CRP < 5 (n = 96)	53 patients	26 patients	14 patients	3 patients	0 patients	0 patients
*p* value*	0.30	**0.01**	0.28	**0.03**	n/a	n/a

Significant values are in [bold].

Patient were frequently on multiple therapies. Twenty-seven patients were on no medications, or only nutritional therapy, at the time of plasma sampling.

*Calculated using a χ^2^ test.

### Metabonomics

NMR-based blood-plasma metabolomic profiles were acquired for all 228 CD patients. Multivariate analysis of these samples identified a subset of patients whose metabolome was markedly characterised by elevated concentrations of ketone bodies (3-hydroxybutyrate, acetone and acetoacetate, Supplementary Fig. 1A). Cross referencing with clinical records revealed these patients had undergone bowel preparation for endoscopy procedure prior to the blood sampling used for NMR analyses. This metabolic perturbation was reflected in the OPLS model, which highlighted ketone bodies as strong discriminants, and overshadowed the importance of other discriminative peaks of the spectrum (Supplementary Fig. 1A, B and C). All 30 patients with documented evidence of bowel preparation for endoscopy prior to plasma collection were therefore excluded from subsequent analyses.

**Figure 2:**
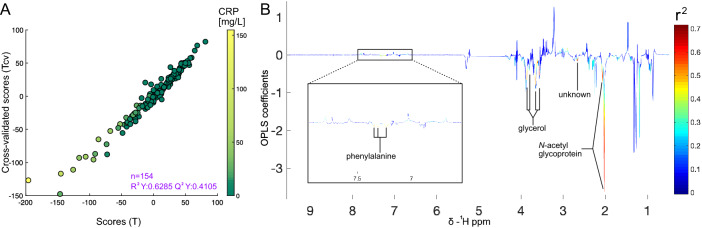
CRP prediction and spectra deconvolution. (**A**) OPLS scores plot. Each point represents one patient spectrum, colour-coded according to CRP levels. Strong correlation between T and Tcv indicates a robust model. (**B**) Loadings plot; colour-scale indicates the correlation magnitude of metabolites with the model scores (r^2^).

Metabolome was regressed against blood CRP readings for the 154 CD patients (R^2^Y = 0.63, Q^2^Y = 0.41; Fig. 2A). The corresponding loadings plot (Fig. 2B) highlights the peaks contributing most to that classification (weight > 0.4). Distinct signals (Table 3) associated with *N*-acetyl glycoprotein (δ 2.01–2.04 ppm), glycerol (δ 3.56, 3.64 ppm), phenylalanine (δ 7.33, 7.38, 7.43 ppm), and an unidentified lipid signal (δ 2.66 ppm) were identified at significantly higher concentration in plasma samples obtained from patients with higher systemic inflammation.

**Table 3:** List of selected signals from the OPLS model.

Peak δ (ppm)	Multiplicity	OPLS weight	variation	Assigned metabolite
2.01	Singlet	0.49	↑	Composite glycoprotein
2.04	Singlet	0.48	↑	Composite glycoprotein
2.66	Multiplet	0.69	↑	Unassigned
3.56	Doublet of doublets	0.64	↑	Glycerol
3.64	Doublet of doublets	0.52	↑	Glycerol
7.33	Multiplet	0.46	↑	Phenylalanine
7.38	Multiplet	0.45	↑	Phenylalanine
7.43	Multiplet	0.49	↑	Phenylalanine

Reported peaks showed an OPLS weight > 0.4. The OPLS weight value represent the R^2 for each metabolites.

### Machine learning classification

An RF model was employed to discriminate patient classes of actively inflamed (n = 58 patients with CRP ≥ 5 mg/L) versus uninflamed (n = 96 patients CRP < 5 mg/L) cases.

The first phase of modelling identified 23.1% percent (8934 datapoints) of the NMR spectrum as informative (Supplementary Fig. 2). On average, the model trained and tested on this fraction of the spectrum was effective in distinguishing the non/inflamed classes (mean F-1 statistic = 0.78 ± 0.05; balanced accuracy = 0.82 ± 0.04; precision = 0.84 ± 0.08 and; recall = 0.74 ± 0.05).

**Figure 3:**
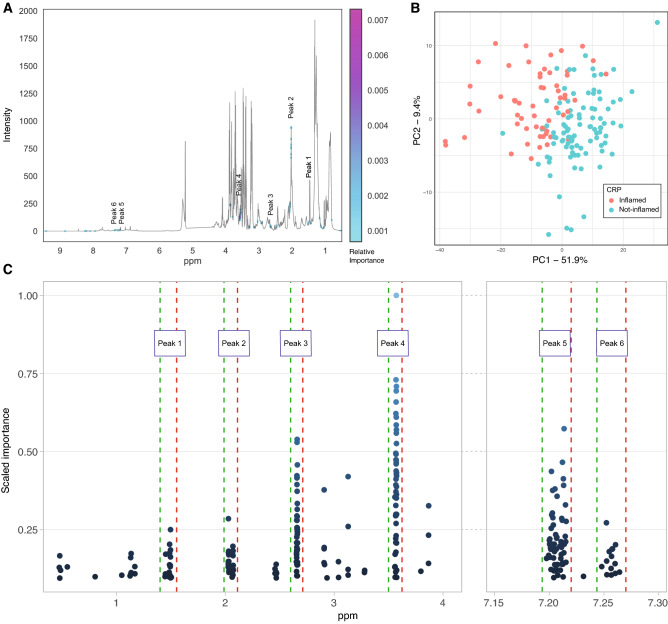
Machine learning classification of patients using NMR data. (**A**) Most informative regions selected by RF model to discriminate patient inflammation status. (**B**) PCA of patient’s spectra using 258 most informative NMR datapoints. (**C**) Distribution of the selected most informative datapoints by their shift δ (ppm) and importance. Green and red dashed lines indicate the start and end of a peak.

Figure 3A shows the regions of the spectrum identified by the RF model as most informative in discriminating patients with and without active inflammation.

PCA modelling of the subset of 258 points evaluated as having a relative importance measure within the top 5th percentile (Fig. 3A; Supplementary Fig. 3) shows reasonable separation between patients according to their inflammation status (Fig. 3B). The distribution of 199 of these points was concentrated within 6 discrete peaks containing ≥ 10 supporting datapoints (Fig. 3C). These six NMR spectrum peaks identified as highly informative in the classification of CD patients with/out active inflammation (Table 4) were compared with the peaks independently identified through OPLS modelling using continuous CRP levels. Reassuringly, peaks 2 and 3 defined by RF modelling recapitulate the OPLS findings of those peaks labelled as GlycA (δ 2.03–2.07 ppm); peak 4 defined by ML corresponds to one (δ 3.56–3.57 ppm) of the two spectral signatures that are noted in the OPLS modelling to depict glycerol; and peaks 5 and 6 in the RF model correspond to the two phenylalanine peaks (δ 7.20–7.26 ppm) as seen in Fig. 2B. Furthermore, the individual data points underlying ML derived peaks 3 and 4 are recognised as having the highest average discriminatory value for classification of inflamed status (average importance of 0.29 and 0.35 respectively) with peak 4 exhibiting the highest mean importance and also containing the single data point with the highest discriminative importance (Fig. 3C, Table 4).

**Table 4:** Machine learning selected NMR peaks.

Peak	Peak min ppm	Peak max ppm	Delta ppm	# of NMR data points	Max importance observed	Average importance observed	PC1 explained variance (%)	PC2 explained variance (%)	Components selected for gene correlation	Identified by OPLS modelling
1	1.45	1.50	0.058	18	0.25	0.14	79.5	14.9	PC1	No
2	2.03	2.07	0.047	21	0.28	0.15	92.4	5.1	PC1, PC4, PC5	Yes (*N*-acetyl glycoprotein)
3	2.65	2.66	0.011	40	0.54	0.25	98.6	0.8	PC1	Yes (unassigned peak)
4	3.56	3.57	0.017	47	**1.00**	**0.39**	82.1	15.2	PC1, PC2	Yes (glycerol)
5	7.20	7.22	0.018	58	0.57	0.22	84.7	2.7	PC1	Yes (phenylalanine)
6	7.25	7.26	0.016	15	0.27	0.16	84.9	4.9	PC1	Yes (phenylalanine)

Significant values are in [bold].

Peaks identified by the RF classifier in the discrimination of CD patients by their CRP status. Reported importance is scaled by the maximum importance observed.

### Metabolomics-genomics integration

Single eigenvectors summarising the six RF peaks significantly discriminating the inflamed and non-inflamed classes after FDR correction (Table 4, Supplementary Fig. 4, Supplementary Fig. 5).

Correlation of each patient’s eigenvector scores for each of the six ML-defined metabolomic peaks against their GenePy gene scores was used to determine any relationship between metabolomic signatures of active inflammation and gene pathogenicity scores. This resulted in sets of nominally significant genes that were either positively or negatively correlated. In order to retain potentially informative biological insight, these genes were then grouped and assessed by direction of correlation (positive, negative, all). These gene sets were then interrogated for enrichment of specific functional pathways that might be useful in interpreting NMR peak signatures (Table 5).

**Table 5:** Enrichment results of gene-peak correlations.

Peak	Enrichment term (term_id)	Term size^a^	Intersection^b^	Correlation set	Adjusted *p* value (SCS)^c^	Enriching genes^d^
1	**Peptide GPCRs (WP:WP24)**	75	11	All	**0.004**	*CCR1, CCR2, CCR5, CCR9, CXCR5, FPR3, GALR1, MC3R, MC4R, OXTR, TRHR*
	Rectum; glandular cells[High] (HPA:0400053)	2641	66	Negative	0.026	Supplementary Table 1
	Peptide GPCRs (WP:WP24)	75	7	Negative	0.033	*CCR1, CCR2, CCR5, CXCR5, FPR3, MC4R, TRHR*
	GPCRs, Class A Rhodopsin-like (WP:WP455)	256	20	All	0.037	Supplementary Table 1
	hSIR2-p53 complex (CORUM:2821)	2	2	Positive	0.050	*SIRT1, TP53*
	SEC23–SEC24 adaptor complex (CORUM:7139)	2	2	Positive	0.050	*SEC23A, SEC24B*
2	Receptor complex (GO:0043235)	379	18	Positive	0.019	Supplementary Table 1
	Regulation of actin cytoskeleton (KEGG:04810)	217	12	Positive	0.028	*BAIAP2, F2R, FGF17, FGFR1, FGFR3, ITGAD, ITGAX, ITGB5, PIK3R1, PIP5K1B, PPP1CC, PPP1R12B*
	RNA polymerase I transcription regulatory region sequence-specific DNA binding (GO:0001163)	8	3	Negative	0.043	*BAZ2A, PIH1D1, RRN3*
	RNA polymerase I core promoter sequence-specific DNA binding (GO:0001164)	8	3	Negative	0.043	*BAZ2A, PIH1D1, RRN3*
3	Intrinsic component of membrane (GO:0031224)	2464	111	All	0.027	Supplementary Table 1
	DTNBP1(1A)-HDAC3 complex (CORUM:7487)	2	2	Negative	0.050	*DTNBP1, HDAC3*
	BKCA-beta2AR complex (CORUM:672)	2	2	Positive	0.050	*ADRB2, KCNMA1*
4	**Intrinsic component of membrane (GO:0031224)**	2464	110	All	**0.003**	Supplementary Table 1
	**Inflammatory bowel disease (KEGG:05321)**	63	7	Negative	**0.003**	*GATA3, IL12B, IL12RB2, IL6, MAF, NFKB1, RORC*
	**Chromatin silencing complex (GO:0005677)**	6	4	All	**0.004**	*BAHD1, BAZ2A, RRP8, SIRT2*
	**Integral component of membrane (GO:0016021)**	2355	105	All	**0.005**	Supplementary Table 1
	Chromatin silencing complex (GO:0005677)	6	3	Positive	0.018	*BAHD1, RRP8, SIRT2*
	Oxidoreductase activity, acting on the CH-NH2 group of donors (GO:0016638)	17	4	Negative	0.035	*GLDC, GLUD1, LOXL4, PNPO*
	BKCA-beta2AR complex (CORUM:672)	2	2	Positive	0.050	*ADRB2, KCNMA1*
5	**ESR-mediated signaling (REACTOME: R-HSA-8939211)**	181	13	Negative	**0.005**	*AGO2, AREG, CXCL12, FKBP5, GNB4, GNG12, GPAM, IGF1R, JUN, PIK3R2, TFF1, TNRC6C, USF2*
	Postsynaptic membrane (GO:0045211)	103	8	Positive	0.022	*CACNG4, CDH2, CNTN2, DAGLA, DBN1, GRIK4, HIP1, KCNMA1*
	RFC complex (CORUM:277–279-2799)	5	3	All	0.050	*RFC1, RFC2, RFC3*
	MSP58-RINT1 complex (CORUM:6314)	5	2	Negative	0.050	*MCRS1, RINT1*
6	Plasma membrane (GO:0005886)	5	105	Positive	0.050	Supplementary Table 1
	Cell periphery (GO:0071944)	2	105	Positive	0.050	Supplementary Table 1
	Intrinsic component of plasma membrane (GO:0031226)	4879	83	All	0.008	Supplementary Table 1
	Protein-arginine deiminase activity (GO:0004668)	4971	3	Negative	0.018	*PADI2, PADI3, PADI4*
	Integral component of membrane (GO:0016021)	1591	112	All	0.018	Supplementary Table 1
	Intrinsic component of membrane (GO:0031224)	5	116	All	0.025	Supplementary Table 1
	SPG3A–SPG33 complex (CORUM:6525)	2355	2	Positive	0.040	*ATL1, ZFYVE27*

Significant values are in [bold].

Enriched terms for genes that positively or negatively correlate with the identified peaks.

^a^The term size indicates the number of genes belonging to a specific term in the relative dataset.

^b^The intersection refers to the number of genes from the correlation analysis that overlaps with a specific term.

^c^SCS correction method embedded in gProfiler2.

^d^The complete list of genes enriching for the named term is reported in the Supplementary Table 1.

Peak 1 is most significantly enriched for ‘g-protein coupled receptors (GPCRs)’ (WP:WP24; *p* = 0.004) when considering all correlated genes, although the signal remains significant when considering only genes that are negatively correlated with inflammation status. Specifically, patients designated as having non-inflamed status exhibit a higher burden of pathogenic variation in genes involved in GPCR signalling. Peak 2 is positively correlated with genes enriched to function within receptor complexes (*p* = 0.02) and regulate actin cytoskeleton (*p* = 0.03).

Metabolomic peak 4, identified by both OPLS and ML modelling to be most strongly associated with inflammation, contains the most significantly enriched functional gene-sets. One hundred and ten genes whose pathogenicity scores are significantly correlated with peak 4 are enriched to function within the ‘intrinsic component of membrane’ (*p* < 0.003; GO:0031224) and its subset-term ‘integral component of membrane’ (105 genes, *p* < 0.003; GO:0016021) (Supplementary Table 1). Interestingly, this is the same functional group identified as correlated with peak 3 suggesting a common biological mechanism might drive both metabolic signatures.

Of particular interest given our clinical cohort, is the set of seven genes correlated with peak 4 identified as enriching for molecular function in ‘inflammatory bowel disease’ (*p* < 0.003; KEGG:05321). This enrichment is specific to negatively correlating genes, indicating that CD patients with active inflammation are more likely to have a *low* burden of pathogenic variation within these genes. The seven nominally correlated genes that combine to define this IBD enrichment term are *GATA3*, *IL12B*, *IL12RB2*, *IL6*, *MAF*, *NFKB1*, *RORC*.

Peak 5 shows a distinct enrichment for the ESR (estrogen signalling receptor) signalling pathway (*p* < 0.005), an important molecular cascade involved in acute and chronic inflammation. Finally, peak 6 shows weaker enrichment for various enrichment terms many of which reflect of plasma membrane function echoed in peaks 3 and 4.

## Discussion

This study combined untargeted metabolomics with whole gene pathogenicity burden scores derived from whole-exome sequencing data from paediatric patients diagnosed with Crohn’s disease. Assimilation of clinical and omic data for patient samples modelled NMR spectra into discrete peaks strongly associated with active inflammation detected in plasma. Individual patient differences in these metabolomic signatures of inflammation appear non-random with respect to functional capacity of genes that elicit the pro-inflammatory immune response for which targeted therapies exist^18,19^.

We used two approaches to determine the regions of patient metabolic spectra most associated with inflammation. Results of both OPLS modelling of continuous CRP and RF modelling of binarised CRP levels, culminated to identify the same regions of the spectrum typically assigned to *N*-acetyl glycoproteins (GlycA), glycerol and phenylalanine. The metabololomic signature of inflammation identified in this study of paediatric CD patients is consistent with that identified in other studies of adult inflammation^20^. GlycA is a composite signal reflecting glycoprotein acetylation of heterogeneous origin^21,22^. Our data independently corroborate this signal as an NMR-derived spectrometric biomarker of systemic inflammation. The same signal was recently highlighted in the context of acute febrile illnesses, chronic inflammatory and autoimmune diseases and found to strongly correlate with CRP, interleukin-6, fibrinogen, serum amyloid A, lipoprotein-associated phospholipase A_2_ and tumour necrosis factor^23,24^. While the data here presented reflects a single snapshot of patient’s inflammation course, previous studies indicated how the GlycA signature might evolve over time^25^—yet with unknown dynamic with respect to CRP—but confirming its role in systemic inflammation^20^.

CRP, produced by hepatocytes in response to IL-6, is a non-specific clinical marker of acute and chronic systemic inflammation. However, its efficacy as a single marker is limited by high inter- and intra-individual variability^26^. Although correlated, it has been suggested that the protein glycan biomarker GlycA and CRP may play distinct roles^27^. CRP levels increase in response to bacteria and intracellular antigens of damaged cells, as an early acute phase response, whereas haptoglobin, α_1_-acid glycoprotein, α_1_-antitrypsin and transferrin, that contribute the most to the GlycA signal, rise later stage of the inflammatory response^28^. GlycA measurement may represent an independent, more stable biomarker of acute response and systemic inflammation.

Regions of the metabolomic spectrum attributed to glycerol and phenylalanine were consistently associated with the inflammation in our CD patients. Phenylalanine is an aromatic amino acid previously linked to metabolic disturbance^29^ and a marker of systemic low grade inflammation possibly arising from liver disfunction, compromised uptake at the blood brain barrier or altered microbiota composition^30^. Glycerol has recently been described as a single molecule systemic biomarker of infection whereby increased glycerol in plasma reflects a metabolic adaptation to intestinal infection, as a provision of sufficient energy for survival^31^. This study provides evidence for a correlation between the NMR glycerol signal and genes known to be involved in the pathogenesis of inflammatory bowel disease. Our data demonstrate increased levels of glycerol in patient’s plasma negatively correlating with individual burden of pathogenic mutations in genes driving pro-inflammatory signalling i.e. patients with wild-type sequence across these genes exhibited a higher metabolic signature of inflammation suggesting a more intact and effective pro-inflammatory response. Despite our data modelling being blind to patient diagnosis, objective assessment of over fifteen thousand genes against the metabolomic signature of inflammation, ‘inflammatory bowel disease’ was amongst the most significantly enriched terms for correlated genes. The seven genes driving this result converge upon pro-inflammatory pathways and extensive data already support their role in IBD. The pathogenesis of Crohn’s disease is multi-factorial, but there appears to be a significant proportion of patients where the underlying genetic risk is related to a hypo-immune response (such as loss-of function variants in *NOD2*)^30^. This concept provides a framework for understanding why low burden of variation in pro-inflammatory ‘IBD’ genes correspond to high glycerol levels. We hypothesise that in these maintained pro-inflammatory pathways, chronic activation occurs due to alternative hypo-immune response to intestinal bacteria, resulting in chronic inflammation and the observed hyper-inflammatory response^32,33^.

Expression of *GATA3*, a mediator of Th2 cytokine response to inflammation is dependent on the p50 subunit of NFKB encoded by *NFKB1*^34^. NFKB is activated by pattern-recognition receptors (PRRs) including the *NOD-*receptors and a master regulator of immune inflammation with an established role in perturbed mucosal inflammation in CD^35,36^. NFKB recruits several pro-inflammatory cytokines in response to microbial stimulation, including IL-12 and IL-23. IL6, in addition to promoting CRP, drives Th17 lineage development—plasticity of which is also influenced by *RORC*^37,38^. *IL12B* is an IBD-associated gene encoding the p40 subunit that is targeted by ustekinumab monoclonal antibody and common to both IL12 and IL23^39,40^. Functional studies in both mice^39^ and human patients^41^ proved how mutations in its coding sequence can alter the inflammatory response through the formation of the IL-12/IL-23 heterodimer. Although, IL12 and IL23 are both implicated in temporally distinct inflammatory responses to intestinal barrier impairment^42^, concurrent implication in our analysis of *IL12RB* that encodes the membrane receptor for the IL12 cytokine might suggest IL12 signalling is driving the inflammatory response in our paediatric cohort. Interestingly GlycA has been previously implicated as a tool for measuring inflammation, and specifically within IBD^43,44^. However this is a non-specific marker and the link to underlying genomic variation requires further investigation.

Future optimisation of the approach applied herein is possible. CRP levels are transient and fluctuate with disease state and treatment. Other than their attendance at routine tertiary clinics, our patients were unselected with respect to their disease state or clinical intervention. It is likely that standardising the patient cohort would further improve power to detect genetic signals. Superior power may be gained by focussing on treatment naïve individuals at point of diagnosis, although such samples can be difficult to attain and remain non-uniform with respect to underlying genetics, steroid and antibiotic use and duration of disease prior to first attendance.

In preparation for endoscopy, patients are restricted to a glucose-containing fluid-only diet from 24-h prior to the procedure and nil-by-mouth for the four hours immediately preceding endoscopy. Our data identified a metabolic signature highly inflated for ketone bodies in these patients that may warrant further clinical consideration.

Our data indicate patients with an altered burden of pathogenic mutation within genes critical to mounting the pro-inflammatory immune response following bacterial exposure, harbour a distinctive metabolomic signature (δ 3.56–3.57 ppm) reflecting inflammatorily active disease. This metabolomic signature and its correlated genes warrant further investigation as biomarkers to stratify CD patients into groups that may respond differently to targeted monoclonal antibodies. While our study focussed on children with a diagnosis of Crohn’s disease, we suggest mutations in these genes are unlikely to represent the primary CD disease trigger in many of these patients, but instead contribute to an individual genomic profile that substantially modulates the inflammatory response and disease progression.

## Supplementary Information

Supplementary Information 1.

Supplementary Information 2.

Supplementary Information 3.

## Data Availability

The datasets generated and/or analysed during the current study are available through direct collaborative agreements, in line with the informed consent gained from all participants.
