# Supplementary material for: Evidence of a genetically driven metabolomic signature in actively inflamed Crohn’s disease
Source: Sci Rep. 2022 Aug 18;12:14101. doi: 10.1038/s41598-022-18178-9 (PMC9388636; doi:10.1038/s41598-022-18178-9)
Supplement: Supplementary file 2 — Supplementary Information 2. [file 41598_2022_18178_MOESM2_ESM.docx]

**Supplementary figures**

**
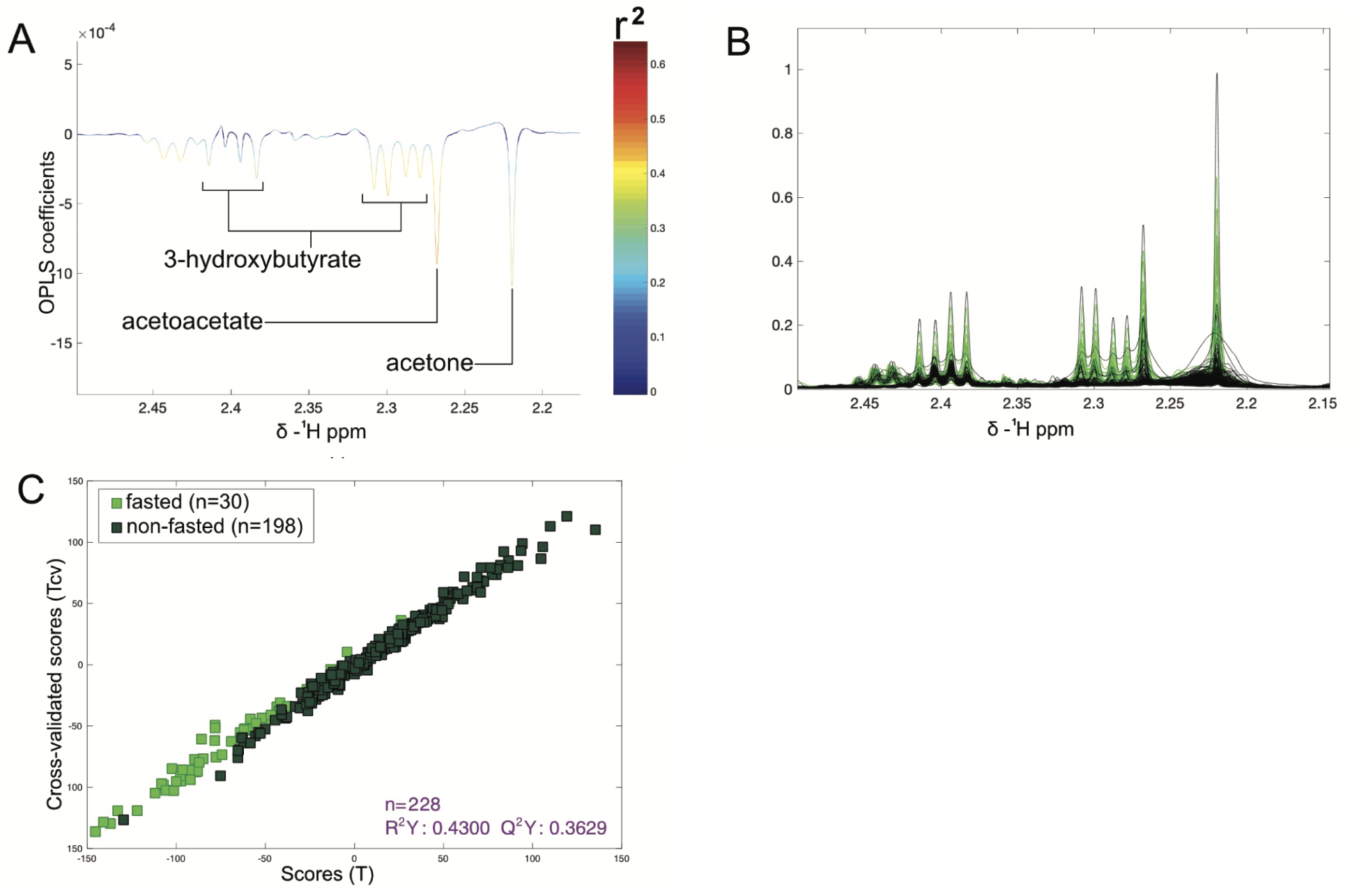
**

Supplementary Figure 1. Fasting effect on plasma metabolic profile. OPLS model for CD cohort (n=228). The regression model was computed using classes vector (0=patients that did go under endoscopy, and therefore were fasted at the time of blood sample was taken, 1=patients that gave out blood with no requirement of fasting). A. Loadings plot. Colour-scale is used to indicate the correlation of the metabolites with the model (r^2^). B. Superimposed spectra of the samples presented in C, colour-coded as follows: light green – fasted, dark green – non-fasted. Scores plot. Each point (score) represents one patient spectrum and is colour-coded as follows: light green – fasted, dark green – non-fasted. Scores (T) are plotted against cross-validated scores (Tcv), which are the scores predicted by the model, therefore strong correlation between T and Tcv indicates a robust model.


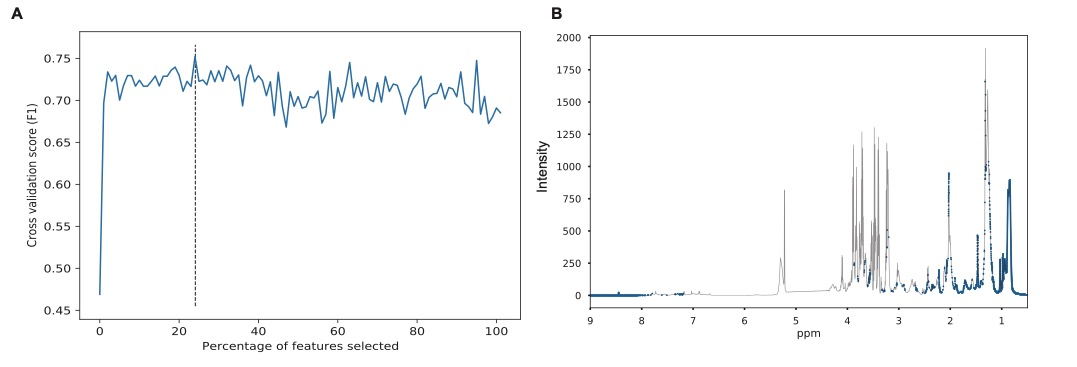


Supplementary Figure 2. Feature selection results. A. Optimal number of features selected by the RFECV. A total of ﻿8943 (23.1% of ﻿38,742 features) datapoints of the NMR are selected as important in classifying patients according to their CRP blood levels. B. Localisation in the NMR spectrum of the selected features.

Supplementary Figure 3. Importance distribution. Distribution of the relative importance assigned to each selected feature by the random forest model. The dashed line indicates the top 5^th^ percentile of the distribution of features with log transformed scaled importance >10^-3^. Importance was scaled with respect to the maximum importance observed.


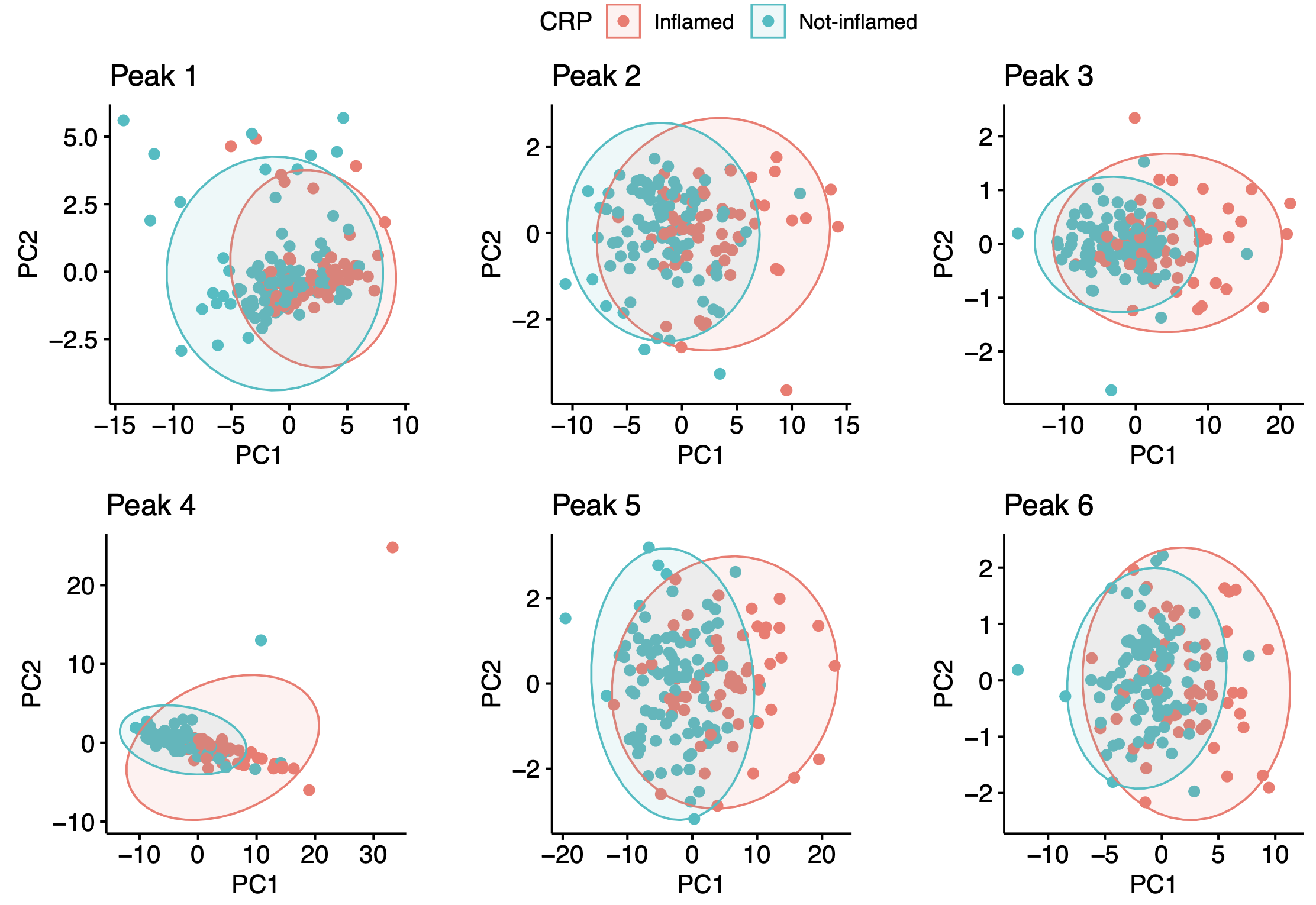


Supplementary Figure 4 PCA decompositions. PCA decompositions and projections of the first two components for the six most important peaks.


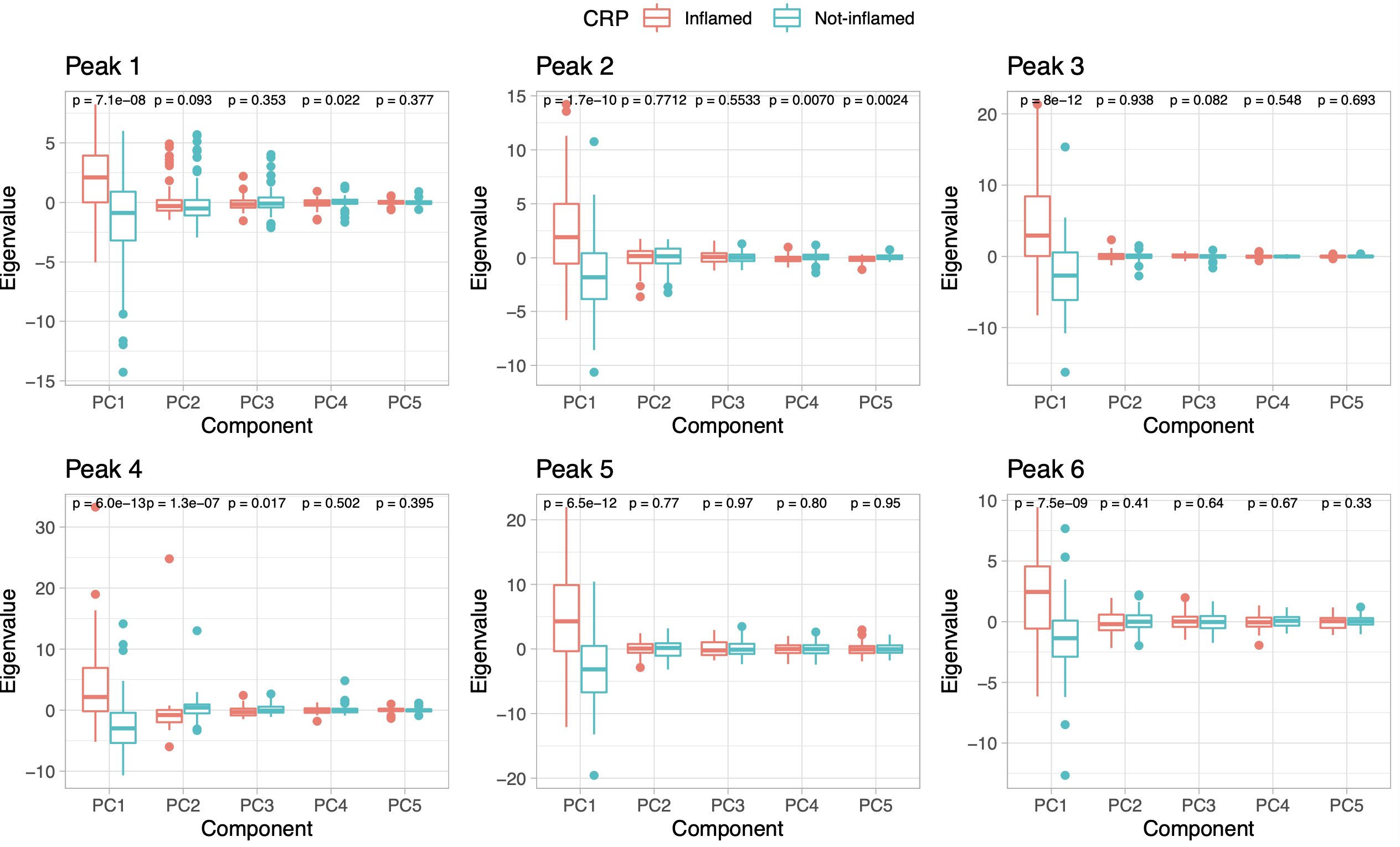


Supplementary Figure 5 Discriminative power of PCs. Discriminative power of the first five principal components (PC) in the classification of CD patients according to their CRP blood levels. P-values were calculated using a Wilcoxon rank sum test and are reported in their non-adjusted form. No significant differences were observed in components not illustrated.
